# Supplementary material for: Tendencies and attitudes towards dietary supplements use among undergraduate female students in Bangladesh
Source: PLoS One. 2021 Apr 9;16(4):e0249897. doi: 10.1371/journal.pone.0249897 (PMC8034729; doi:10.1371/journal.pone.0249897)
Supplement: S1 File — (DOCX) [file pone.0249897.s001.docx]

**Written consent of the respondent**

**Dietary supplement use among undergraduate female students in public and private universities located in Chittagong, Bangladesh: prevalence, opinions, and attitudes**

Observer:______________________

**Purpose of the study:** This questionnaire is set for exploring the dietary supplement use among undergraduate female students in Chittagong City. Factors associated with their age group, sex, profession, localities, environment is considered very profoundly.

**Confidentiality:** Privacy policy for individual patients will be preserved with cautions and commitment. All questions are needed to answer but attendant’s freedom is as well recognized. This questionnaire will be used only for research purpose

Signature and Date: _____________
