# Supplementary material for: Tendencies and attitudes towards dietary supplements use among undergraduate female students in Bangladesh
Source: PLoS One. 2021 Apr 9;16(4):e0249897. doi: 10.1371/journal.pone.0249897 (PMC8034729; doi:10.1371/journal.pone.0249897)
Supplement: S2 File — (DOCX) [file pone.0249897.s002.docx]

**Title: Dietary supplement use among undergraduate female students in public and private universities located in Chittagong, Bangladesh: prevalence, opinions, and attitudes**

[This questionnaire is set for exploring the dietary supplement use among undergraduate female students in Chittagong City. Factors associated with their age group, sex, profession, localities, environment is considered very profoundly. Privacy policy for individual patients will be preserved with cautions and commitment. All questions are needed to answer but attendant’s freedom is as well recognized. This questionnaire will be used only for research purpose]

**Questionnaire**

**Part A: Demographic information**

The following statements relate to your perceptions about“**Dietary supplement use among undergraduate female students in public and private universities located in Chittagong, Bangladesh: prevalence, opinions, and attitudes”**. Please Mark (√) the extent to which you suit with each statement.

| **Demographic information:** | | |  |  |  |  |  |  |  |  |
| --- | --- | --- | --- | --- | --- | --- | --- | --- | --- | --- |
| **Age** |  | 18-23 years |  | 24-26 years |  | 27-30  years |  |  |  |  |
| **Study year** |  | Ist year |  | 2^nd^ year |  | 3^rd^ year |  | F 4^th^ year |  |  |
| **Ethnic origin** |  | Bangladeshi |  | Tribal |  | Non Tribal Bengali |  |  |  |  |
| **Current marital status** |  | Married |  | Unmarried |  | Divorced |  |  |  |  |
| **Residence** |  | Living with family |  | Living alone (University accommodation) |  | Living alone (Self accommodation,) |  |  |  |  |
| **Siblings** |  | Between 1 and 2 siblings |  | Between 3 and 5 siblings |  | Between 6 and 8 siblings |  | More than 8 siblings |  | No siblings |
| **Dietary supplement use in the last month** |  | Daily |  | Weekly |  | Never |  | Not sure |  |  |
| **Name of the subject** |  | Pharmacy |  | BBA |  | EB |  | Law |  | CSE |
|  |  | EEE |  | Quranic Science |  | Dawa |  | Hadith |  |  |
|  |  | English |  | Others |  |  |  |  |  |  |
| **University type** |  | Private |  | Public |  |  |  |  |  |  |

**Part B:**

1. **Suffering any major illness (if yes) please mark**

| Any major illness |  | Do not suffer from any illness |  |  |  |  |  |  |  |  |
| --- | --- | --- | --- | --- | --- | --- | --- | --- | --- | --- |
| Suffer from a major illness |  | Depression, |  | Hypertension |  | Diabetes mellitus |  | Sickle cell anemia |  | Thalassemia |
|  |  | Asthma |  | Rheumatoid arthritis |  | Psoriasis |  | Ulcer |  | Irregular manustration |
|  |  | Migraine |  | Obesity |  | Epilepsy |  |  |  |  |
|  |  |  |  |  |  |  |  |  |  |  |

1. **Reasons for use of dietary supplement**

Please indicate (√) how much you agree or disagree with each of the following statements.

| Strongly Disagree | Moderately Disagree | Slightly Disagree | Neither Agree Nor Disagree | Slightly Agree | Moderately Agree | Strongly Agree |
| --- | --- | --- | --- | --- | --- | --- |
| 1 | **2** | **3** | **4** | **5** | **6** | **7** |

| Itemcode |  | 1 | 2 | 3 | 4 | 5 | 6 | 7 |
| --- | --- | --- | --- | --- | --- | --- | --- | --- |
|  | **Reasons for use of dietary supplement (RFDS)** |  |  |  |  |  |  |  |
| RFDS 1 | Physician recommendations |  |  |  |  |  |  |  |
| RFDS 2 | General health and well being |  |  |  |  |  |  |  |
| RFDS 3 | For weight gaining |  |  |  |  |  |  |  |
| RFDS 4 | For energy source |  |  |  |  |  |  |  |
| RFDS 5 | Immune booster |  |  |  |  |  |  |  |
| RFDS 6 | Increase performance/sports |  |  |  |  |  |  |  |
| RFDS 7 | To control hair fall and skin care |  |  |  |  |  |  |  |
| RFDS 8 | Increase endurance/body building |  |  |  |  |  |  |  |
| RFDS 9 | Memory enhancer |  |  |  |  |  |  |  |
| RFDS 10 | Other reasons (pregnancy-induced anemia and fatigue) |  |  |  |  |  |  |  |
| RFDS 11 | No reason mentioned |  |  |  |  |  |  |  |

1. **Types of dietary supplement**

Please indicate (√) how much you agree or disagree with each of the following statements.

| Strongly Disagree | Moderately Disagree | Slightly Disagree | Neither Agree Nor Disagree | Slightly Agree | Moderately Agree | Strongly Agree |
| --- | --- | --- | --- | --- | --- | --- |
| 1 | **2** | **3** | **4** | **5** | **6** | **7** |

| Itemcode |  | 1 | 2 | 3 | 4 | 5 | 6 | 7 |
| --- | --- | --- | --- | --- | --- | --- | --- | --- |
|  | **Types of dietary supplement (TDS)** |  |  |  |  |  |  |  |
| TDS 1 | Multivitamins alone or in combination with others |  |  |  |  |  |  |  |
| TDS 2 | Ginseng and Gingko biloba |  |  |  |  |  |  |  |
| TDS 3 | Omega 3 fatty acid |  |  |  |  |  |  |  |
| TDS 4 | Whey protein |  |  |  |  |  |  |  |
| TDS 5 | Calcium |  |  |  |  |  |  |  |
| TDS 6 | Other supplements (prescription and natural products) |  |  |  |  |  |  |  |
| TDS 7 | No supplements used |  |  |  |  |  |  |  |

1. **Cost of dietary supplements per month in Taka**

Please indicate (√) how much you spend in each month

|  | Tk 10-500 |  | Tk 501-1000 |  | Tk 1001-5000 |  | Tk 5001-10000 |  |  |
| --- | --- | --- | --- | --- | --- | --- | --- | --- | --- |

1. **Adverse reactions experienced from dietary supplement**

Did you experience any adverse reactions?

|  | yes |  | No |  |
| --- | --- | --- | --- | --- |

**If yes, what were they?**

|  | Nausea,vomiting, and diarrhea |  | Confusion, headaches, and vertigo |  | Hair fall |  | Rapid Weight Gain |  | Others |  |  |
| --- | --- | --- | --- | --- | --- | --- | --- | --- | --- | --- | --- |

1. **Opinions and attitudes regarding dietary supplement**

Please indicate (√) how much you agree or disagree with each of the following statements.

| Strongly Disagree | Moderately Disagree | Slightly Disagree | Neither Agree Nor Disagree | Slightly Agree | Moderately Agree | Strongly Agree |
| --- | --- | --- | --- | --- | --- | --- |
| 1 | **2** | **3** | **4** | **5** | **6** | **7** |

| Itemcode |  | 1 | 2 | 3 | 4 | 5 | 6 | 7 |
| --- | --- | --- | --- | --- | --- | --- | --- | --- |
|  | **Opinion and attitudes of dietary supplement (OADS)** |  |  |  |  |  |  |  |
| OADS 1 | It prevent chronic illness if used regularly |  |  |  |  |  |  |  |
| OADS 2 |  |  |  |  |  |  |  |  |
| OADS 3 | Safe with minimal risk of adverse effects |  |  |  |  |  |  |  |
| OADS 4 | Essential for everyone regardless of age |  |  |  |  |  |  |  |
| OADS 5 | Prevents cancer |  |  |  |  |  |  |  |
| OADS 6 | Important for health and general well-being |  |  |  |  |  |  |  |
| OADS 7 | Use only as per physician recommendation/ harmful if not used properly |  |  |  |  |  |  |  |
| OADS 8 | Necessary for all ages |  |  |  |  |  |  |  |

1. **Dietary supplements are good for health**

|  | Agree |  | Disagree |  | Do not Know |
| --- | --- | --- | --- | --- | --- |

1. **Do you personally recommend use of dietary supplements to others**

|  | Yes, I always recommend |  | Yes, only when doctors recommend |  | Not at all |
| --- | --- | --- | --- | --- | --- |

1. **Dietary supplement product information**

**If you know (**Please indicate (√) **any information related to diatery suppliments that you have been used or using**

|  | Brand name |  | Generic Name |  | Both |  | Do not Know |
| --- | --- | --- | --- | --- | --- | --- | --- |
