# Supplementary material for: Tendencies and attitudes towards dietary supplements use among undergraduate female students in Bangladesh
Source: PLoS One. 2021 Apr 9;16(4):e0249897. doi: 10.1371/journal.pone.0249897 (PMC8034729; doi:10.1371/journal.pone.0249897)
Supplement: Attachment — Submitted filename: Main Manuscript Final_Plos One 27122020.docx [file pone.0249897.s004.docx]

**Research Article**

**Tendencies and attitudes of young students towards dietary supplements in Bangladesh: female students are more inclined**

Ishrat Jahan^1^, Abul Bashar Mohammad Neshar Uddin^1^, A.S.M. Ali Reza^1,3^, Md. Giash Uddin^2^, Mohammad Shahadat Hossain^1^, Mst. Samima Nasrin^1,3^, Talha Bin Emran^4^, Md. Atiar Rahman^3*^

^1^Department of Pharmacy, International Islamic University Chittagong, Kumira, Chittagong-4318, Bangladesh.

^2^Department of Pharmacy, Faculty of Biological Sciences, University of Chittagong, Chittagong-4331, Bangladesh

^3^Department of Biochemistry and Molecular Biology, University of Chittagong, Chittagong-4331, Bangladesh.

^4^Department of Pharmacy, BGC Trust University Bangladesh, Chittagong-4381, Bangladesh

**Running Title:** Use of Dietary supplements by female students

***Corresponding author:** Md. Atiar Rahman PhD, Professor, Department of Biochemistry and Molecular Biology, University of Chittagong, Chittagong-4331, Bangladesh, Tel: +88-031-2606001-10, Extension- 4334, Fax: +88-031-726310, E-mail: atiar@cu.ac.bd

**Abstract**

**Background**

Dietary supplements (DS) are nutraceuticals that improve the overall health and well-being of individuals and reduce the risk of disease. Evidence indicates a rising prevalence of the use of these products worldwide especially among undergraduate students. The study aims to investigate the trends and inclination of Bangladeshi students towards dietary supplements (DS).

**Methods**

A three-month cross-sectional survey was conducted in undergraduate students in Chittagong, Bangladesh using a pre-validated dietary supplement questionnaire (DSQ). The study was carried among the private and public university students of different disciplines in Chittagong to record their prevalent opinions and attitudes toward using DS. The results were documented and analyzed by SPSS version 22.0.

**Results**

Eighty four percent of respondents answered the survey questions. The prevalence of DS use was higher among undergraduate female students. The female students significantly cited general health and well-being (n=102, 11.0%) and physician recommendation (n=101, 10.9%) as a reason for DS use. Majority of the students (n=817, 88.3%) used DS cost monthly between USD 0.12 to USD 5.90. Most of the students (n=749, 81.0%) agreed on the beneficial effect of DS and a significant portion (n=493, 53.3%) recommended for a regular use of DS. Highly prevalent use of dietary supplements appeared in Chittagonian undergraduate female students. They were tremendously positive in using DS. The results demonstrate an increasing trend of using DS especially by the females for both nutritional fortification and amelioration from diseases.

**Conclusion**

The prevalence of DS users was much higher among private university students than among public universities. Likewise, maximal prevalence is noted in health-related departments such as pharmacy compared to other non-health-related departments. Students preferred brand products, had positive opinions and attitudes towards DS.

**Keywords:** Dietary supplement, Undergraduate female students, Prevalence, Awareness, Attitudes, Bangladesh.

**Introduction**

Dietary supplement (DS) is a manufactured product containing one or more than one dietary elements such as vitamins, minerals, herbs or botanicals products, fatty acids, proteins purposive to supplement the diet (ref). DS provides nutrients either extracting from food or synthetic sources administered through the mouth as a tablet, capsule, or pill. It also contains other constituents that may not be therapeutically effective or essential for life, such as polyphenols or plant pigments. In many countries, DS’s are deliberated as a subset of foods and controlled consequently (1). DS’s are commonly used by adults to progress or maintain health as well as disease recovery (2, 3). The use of DS is gradually increasing over time over the world (3). Around 68% of US adults is reported to use DS in different forms. During 2007-2010 in the United States (US), it was assessed that 23% of the supplements was suggested by the health care professional (4). Another US investigation through NHANES (1999–2012) reported that nearly 52% of responders from the US had an uninterrupted DS consumption which is found as diverse testaments in US and Europe for extensive consumption (5). Bangladesh is located in South Asia, where the use of DS among individuals is very common. During 2018, an assessment showed that approximately 41.3% of participants used the DS at least once in the previous year. The number of female users was 45%, which was higher than that of male users. Additionally, 33% of the users started taking supplements without any prescription or consultation healthcare professionals such as pharmacists consisting vast knowledge on the safer and effective uses of DS. DS’s using rate is less in good health people than in poor health people. The aims, perception and opinions of using DS varies in different age and professional groups while kids are given DS’s with the suspicion of nutritional insufficiency (5).

The college and undergraduate students feel they need extra nutrients from DS to compensate their energy deficit for extracurricular activities, study, or part-time jobs. Researchers reported 66% of the five US university students consume DS (6). 63% of female participants from 16 US medical colleges use DS while male DS consumers were lesser than female (7). Another study showed that 76.6% of Saudi female students love to use DS (8). An insignificant difference between male and female DS uses in Japan and 56% of Australian DS using students in 2015 are reported in literature (9). Unfortunately, no such study has been conducted for Bangladeshi students. This is the first study which conducted to explore the prevalence, user’s opinions and attitudes towards the use of DS among undergraduate female students in Chittagong city of Bangladesh.

**Materials and Methods**

**Study design and duration**

A cross-sectional study was planned and conducted in undergraduate female students for an epoch of three months. All the participants of this study were females who had been randomly selected between March 2018- May 2018. The inclusion criteria were: willingness to participate in the study, age >18 years, ability to understand English or Bengali, and participants with previous experience of using dietary supplements. Participants who did not match the above criteria were excluded from the study. In addition, dropout students also excluded from the study. Finally, incompletely or incorrectly filled questionnaires were also not included.

**Study site**

The study was carried out in public and private universities located in Chittagong, Bangladesh. Chittagong division is geographically the largest of the eight administrative divisions of Bangladesh including seven public and six private universities.

**Sample size calculation**

Sample size was calculated by using Raosoft, Inc. (USA) ((10)). Present survey involved 925 respondents who were undergraduate female students from listed private and public universities.

**Study population**

This cross-sectional study included 925 students randomly chosen from the departments of Pharmacy, Bachelor of Business Administration, Economics and Banking, Law, Computer Science and Engineering, Electronics and Electrical Engineering, Quranic Science, Dawa and Islamic Studies, Hadith, English Language and Literature from different universities.

**Purposes of the study**

The main purpose of our survey was to explore the dynamics, of using dietary supplements by the undergraduate females, involving the respondent’s choice, age, discipline, nature of institution, . The survey was directed among several age ranges of 18-23, 24-26, and 27-30 years. The cause of the ordination is the education period. The inclusion of undergraduate students usually occurs in 18 years, and duration varies from 4-5 years in different disciplines. Second important objective was to inquire the differences of using DS with the variation of demography, particular opinion, attitudes, disciplines and ages. Expense regarding dietary supplement usage was recorded and indicated by the BDT (Bangladeshi Taka).

**Research instrument development**

The authors prepared a questionnaire with the title of “the dietary supplement questionnaire (DSQ)”. It consists of several multiple-choice questions. The DSQ was further polished by a group of specialists led by two assistant professors. The DSQ was subdivided into two parts: Part A contains questions in respect of demographic information like age, study year, ethnic region, current marital status, residence, siblings, use of dietary supplement in the last month, name of the subject, and university type. Part B included the questions where respondents were asked on their major illness, reasons for using DS, types of DS they used, their affordability (cost/month in BDT), adverse reactions experienced from DS, opinions, and attitudes. Furthermore, they were asked about their compliance regarding DS's role in health and if they would personally recommend using DS to others. They were also inquired whether they know about DS they had used or currently using. On request from the corresponding author, the DSQ is available.

**Pilot study and accessibility of the DSQ**

A pilot study of the DSQ was performed by distributing the questionnaire among 100 undergraduate female students belonging to different universities' disciplines. A 93% of response rate proved that there were no troubles in understanding the questionnaire. At this point, the DSQ was considered suitable to use.

**Data analysis**

The data were analyzed by statistical software Statistical Package for Social Science (SPSS, version 22.0, IBM Corporation, NY). Frequency counts (N) and percentages (%) were introduced to represent the demographic information, opinions, and attitudes. The chi-square test analyzed the observation of interconnections between student characteristics, i.e., independent variables (IV) and DS study variables, i.e., dependent variables (DV). For documenting interrelation between IV and DV, Spearman’s rank correlation was employed. Regression analysis was conducted to determine different relationships between DS use and demographic characteristics of students.

**Participant consent and ethical consideration**

The survey was permitted to the institutional review committee of International Islamic University Chittagong (ref: P&D-147/13-19). Participants were known to purposes, protocol, and importance of the survey. The participation was deliberate except any influence. Before supplying the questionnaire, an informed written consent was searched from students.

**Results**

The reflection of the respondents on the use of DS has been summarized as follows:

**Demographic information of the respondents**

The demographic information of the participants is shown in **Table 1**. The majority (n=823, 89.0%), out of 925 respondents, in our study participated from private universities. More than three and a half quarter of the students belonged to the age group 18-23 years (n=859, 92.9%). The majority of students were in 4th year (n=343, 37.1%), followed by 3rd year (n=215, 23.2%), during the study years. Almost all of the respondents of the study were found to be unmarried (n=885, 95.7%). Majority of the students were living with family (n=757, 81.8%) and were between 1 and 2 siblings (n=422, 45.6%). These data were contributed by the department of Pharmacy (25.3%), Bachelor of Business Administration (11%), Economics and Banking (14.4%), Law (14.7%), Computer Science and Engineering (12.8%), Electrical and Electronics Engineering (7.5%), Quranic Science (3.4%), Dawa (1.7%), Hadith (1.3%), English Language and Literature (8%) of different universities shown in **Figure 1**.

**Suffering of the respondents from major illness**

The detailed information for suffering of the respondents from major illness (if there is) is summarized in **Table 2**. Collectively, most of the students suffer from depression (n=134, 14.5%) leading to hypertension (n=96, 10.4%). Besides, less than two-quarters of the students did not have any major illness (n=399, 43.1%).

**Etiologies of using dietary supplements**

The detail of the reasons for consuming DS’s is presented in **Table 3**. The greater portion of the respondents took DS to improve their general health and well-being (n=102, 11.0%) while others consumed with physician’s recommendation (n=101, 10.9%). There was no rationale (n=124, 13.4%) for the use of DS amongst those who disagreed most strongly.

**Types of dietary supplement used**

A mixed-response was received on the type of dietary supplement the respondents sought out. Strongly agreed participants (10.4-10.9%) choose DS to be fortified with whey protein (n=101, 10.9%), followed by omega 3 fatty acid. Strongly disagreed respondents ranging from 13.0-13.7% who sought DS to receive calcium (n=127, 13.7%). In contrast, less than two-quarters of the participants disagreed to use no supplement. The details of the types of DS’s uses are presented in **Table 4**.

**Expenditure for dietary supplements per month**

A brief summary on monthly cost of DS’s is presented in Table 4. The monthly cost of DS’s significantly differed among the participants. More than three and a half (n=817, 88.3 %), with DS spent monthly between BDT 10 (USD 0.12) and USD 500 (USD 5.90) participants. BDT 5000 (USD 58.99) and BDT 10 (USD 0.12) were the gross and minimum expenses involved in a month.

**Adverse reactions experienced from dietary supplements**

The details of an adverse reaction are presented in **Table 5**. The participants were asked if any adverse reactions to the use of DS had occurred. More than 59.0% of the respondents (n=546) experienced no adverse reactions over DS sue. Nausea, vomiting and diarrhea (n=75, 8.1%) were the common adverse reactions for those who experienced unwanted effects. Confusion, headaches, and vertigo were encountered by a very insignificant number of respondents (n=09, 1.0%).

**Pharmacy student’s opinions and attitudes regarding dietary supplement use**

Detailed information on the views and attitudes of pharmacy students about DS usage is provided in **Table 7**. The data on the responses of pharmacy students using DS were close to each other. More than 14.1% (n=130) of the respondents strongly disagreed to use DS in all ages while 11.5% (n=108) strongly agreed on the same regardless of ages Mentionable number of participants agreed to use DS for cancer prevention (n=106, 11.5%).

**Dietary supplements are good for health**

The health specifics of DS use are shown in **Table 6**. More than three quarters and a half of students accepted that the use of DS is good for health (n=749, 81.0%).

Additionally, less than a quarter of students did not know whether its use is good for health or not (n=139, 15.0%).

**Personal advice to others when using dietary supplements**

Details of personal recommendations relating to the use of DSs by others are given in **Table 7**. The students were asked about their thoughts concerning DS's use. Almost half of the students (n=493, 53.3%) recommended the daily use of DS while 37% of doctors did the same.

**Dietary supplement product information**

The respondents were asked whether they were aware of DS product information. Very significant part of the students (n=312, 33.7%) recognized the brand name of DS while a smaller fraction sensed either brand or generic name of the product (n=127, 13.7%). The DS product attributes are shown in **Table 8**.

**Association of demographic variables with opinions and attitudes**

The association of demographic variables with opinions and attitudes are attributed in **Table 11**. The dependable variable (DV) of student opinion towards DS’s use good for health was associated with: age (p-value = 0.927), study year (p-value = 0.677), current marital status (p-value = 0.064), residence (p-value = 0.382), siblings (p-value = 0.832), university type (p-value = 0.735). Moreover, the DV of student opinion towards encouraging the use of DS was associated with: age (p-value = 0.507), study year (p-value = 0.510), current marital status (p-value = 0.490), residence (p-value = 0.097), siblings (p-value = 0.094), university type (p-value = 0.928).

**Multinomial regression analysis**

Multinomial regression analysis on detail results is presented in **Table 12**. Multinomial logistic regression (MLR) was used to interpret the odds ratio for dependent variable (DV) of, “recommending dietary supplement use”. And the “age” was considered as an independent variable (IV) where “age variation” was fixed as a categorical IV. Student’s attitudes towards recommending DS use were taken as DV. The parameter of IV, i.e. “age: 24-26 years”, was redundant and therefore set to zero (0). The regression analysis stated that the odds of, “always recommending DS use” increases with every year-wise increase in age (OR’ 0.982). And also Students of “Age: 18-23years” were more likely to “recommend DS use” as compared to students of “Age: 24-26years” (OR’ 0.982).

**4. Discussion**

The use of dietary supplements is increasingly growing because they are easily accessible, affordable and consumer-compliant. The current survey was conducted to determine the prevalence, opinions and attitudes towards the use of dietary supplements of undergraduate female students at public and private universities in Chittagong, Bangladesh. The study was prevailed with the Bangladeshi unmarried private-university-female students of 18-23 years. The study revealed that the use of DS increases over the current years. Several previous studies demonstrated the relationship between higher educational level and DS use. A significant straight relation between higher education level and dietary supplement usage among the full-aged population of France was displayed by Pouchieu and colleagues (11). Likewise, a survey accomplished by Mileva-Peceva *et al.* indicated that highly educated females consume an extremely significant amount of vitamins and /or mineral food supplements (12). Hence, our results are consistent with the above findings.

The majority of the female students, living with families, suffer from major illnesses, i.e., depression, hypertension, diabetes mellitus, sickle cell anemia, thalassemia, asthma, rheumatoid arthritis, migraine, and others. Our results displayed that DS usage is higher in females with high education, which is also coherent with former studies. The National Health and Nutrition Examination Survey (NHANES) 2003-2006 displayed, 61% of DS users passed high-school while only 37% of those were not able to do so (13). NHANES 1999-2000 reported 62% of DS users with more than high-school education, 48% of those with high-school education and just 35% of those with less than high-school education (14). The highest prevalence of our sample was in the 18-23 age groups and the minimum in the 24-26 age range. Among female students of private universities, more popular use of DS's was observed. The reasons behind this could be the socioeconomic situation and services. These students typically belong to a relatively loftier socioeconomic condition and are aligned with support programs for students, healthcare facilities, and ease of consultation on physical activity, social and individual problems, and perceived dietary supplements. Whereas, most of the students enrolled in the public universities belong to low-socioeconomic status and these universities have low tuition fees (15). Hence, the prevalence of DS use in public universities is lower than in private universities.

The female students were asked about the reasons behind the use of the DS. Students mentioned various reasons, such as recommendations from physicians, general health and well-being, weight gain, energy source, immune booster, performance or sports increase, hair fall and skin care control, endurance or bodybuilding increase, memory enhancer, other reasons involve pregnancy-induced anemia and fatigue while few others mentioned no reason. In our study, all the mentioned reasons for the usage of DS’s were so close to each other. Various types of DS including multivitamins alone or in conjunction with Ginseng and Gingko Biloba, Omega 3 fatty acid, whey protein, Calcium, other supplements (prescription and natural products) and non-supplements used by the participants. Previous studies showed that protein supplementation is verily common among students as it improves physical performance (16, 17) whereas Omega 3 fatty acid may increase sleeping attribute and decrease depression and anxiety (18). Our study indicates that a small portion of the students suffered from adverse reactions by using DS, in most cases, they struggled with nausea, vomiting and diarrhea which are mostly related to the previous studies reported in the literature (19-21). The monthly expense of DS’s differed notably among the female students. Most of them (88.3%) indicated a monthly expense between Taka 10-500. Female students preferred brand products rather than generics. Personal preferences are probably the reason behind this. Studies displayed that brand products are trusted to possess preferable quality; therefore, brands may be preferred by the individual (16, 22). In a previous study it is also reported that students spent less than 1 to 3 USD per month (21). This study observed pharmacy student's opinions and attitudes regarding DS use. They give various statements from which the most considerable portion of students strongly disagreed with the statement that they only used DS as per the physician's recommendation. Moreover, the largest portion of students strongly agreed to the statement that they used DS as essential for everyone regardless of age. All the values of given statements for pharmacy students' opinions and attitudes regarding DS use were so close to each other.

In previous study most of the students also agreed to use DS as per the physician’s recommendation (21). But in another study in Lebanon most of the participants believed that they should use DS’s recommendation of friends, peers, and relatives (23). This study searched the opinions and attitudes of undergraduate female students about DS. The demographic variables of the age, study year, ethnic origin, current marital status, residence, siblings, and university type were associated. Most of the female students trusted that DS's are good for health. An insignificant number of them disagreed with this, and the remaining students opined that they do not know regarding this. Several studies documented DS users having some positive attitude along with their health (24). They were more likely to be prosperous, health sensible, less likely to smoke, and probably consume more hygienic food (25). Various studies about the demographic features of DS's consumers also revealed that they were chiefly high educated females with young age (20-39 years) (24, 26). These consequences were compatible with our study outcomes.

**Conclusions**

To sum up, the survey gives details about the highly prevalent usage of dietary supplements among undergraduate female students in Chittagong, Bangladesh. Prevalence was so much higher in students of private universities as compared to students of public universities. Likewise, maximal prevalence is indicated in health-related departments such as pharmacy compared to other non-health-related departments. The limitation of our study was the non-inclusion of male students. To realize DS's usage and how opinions change in identical populations according to gender would have determined by including male students. Therefore, the authors recommend leading the study in the male population.

**Acknowledgements**

The authors are thankful to the Department of Pharmacy, International Islamic University Chittagong, Bangladesh, for research facilities and other logistic supports. The authors are also thankful to Mr. Mohammed Moniruzzaman Bhuiyan, Department of Statistics, University of Chittagong, Chittagong-4331, Bangladesh, for his generous help in the statistical analysis.

**Availability of data and materials**

The datasets used and/or analyzed during the current study are available from the corresponding author on reasonable request.

**Conflict of interest**
Authors declared that they have no conflict of interest.

**Author contributions**

ASMAR and MSN arranged the whole facilities for the research and supervised the research. IR collected the data with the help of ABMNU and MSH. MGU performed the statistical analysis. TBE performed the data analysis and manuscript correction. MAR thoroughly revised the manuscript and interpreted the data.

**References:**

1.	Halsted CH. Dietary supplements and The American Journal of Clinical Nutrition. Oxford University Press; 2000.

2.	Kantor ED, Rehm CD, Du M, White E, Giovannucci EL. Trends in Dietary Supplement Use Among US Adults From 1999-2012. Jama. 2016;316(14):1464-74.

3.	Bailey RL, Gahche JJ, Lentino CV, Dwyer JT, Engel JS, Thomas PR, et al. Dietary supplement use in the United States, 2003-2006. The Journal of nutrition. 2011;141(2):261-6.

4.	Bailey RL, Gahche JJ, Miller PE, Thomas PR, Dwyer JT. Why US Adults Use Dietary Supplements. JAMA Internal Medicine. 2013;173(5):355-61.

5.	Sato Y, Yamagishi A, Hashimoto Y, Virgona N, Hoshiyama Y, Umegaki K. Use of dietary supplements among preschool children in Japan. J Nutr Sci Vitaminol (Tokyo). 2009;55(4):317-25.

6.	Lieberman HR, Marriott BP, Williams C, Judelson DA, Glickman EL, Geiselman PJ, et al. Patterns of dietary supplement use among college students. Clinical Nutrition. 2015;34(5):976-85.

7.	Spencer EH, Bendich A, Frank E. Vitamin and Mineral Supplement Use among US Medical Students: A Longitudinal Study. Journal of the American Dietetic Association. 2006;106(12):1975-83.

8.	Alfawaz H, Khan N, Alfaifi A, Shahrani FM, Al Tameem HM, Al Otaibi SF, et al. Prevalence of dietary supplement use and associated factors among female college students in Saudi Arabia. 2017;17(1):116.

9.	Wiltgren AR, Booth AO, Kaur G, Cicerale S, Lacy KE, Thorpe MG, et al. Micronutrient supplement use and diet quality in university students. Nutrients. 2015;7(2):1094-107.

10.	<http://www.raosoft.com/sample> size.html.

11.	Pouchieu C, Andreeva VA, Péneau S, Kesse-Guyot E, Lassale C, Hercberg S, et al. Sociodemographic, lifestyle and dietary correlates of dietary supplement use in a large sample of French adults: results from the NutriNet-Sante cohort study. 2013;110(8):1480-91.

12.	Mileva-Peceva R, Zafirova-Ivanovska B, Milev M, Bogdanovska A, Pawlak RJP. Socio-demographic predictors and reasons for vitamin and/or mineral food supplement use in a group of outpatients in Skopje. 2011;32(1):127-39.

13.	Bailey RL, Gahche JJ, Lentino CV, Dwyer JT, Engel JS, Thomas PR, et al. Dietary supplement use in the United States, 2003–2006. 2011;141(2):261-6.

14.	Radimer K, Bindewald B, Hughes J, Ervin B, Swanson C, Picciano MFJAjoe. Dietary supplement use by US adults: data from the National Health and Nutrition Examination Survey, 1999–2000. 2004;160(4):339-49.

15.	Abbas AJP. Evidence based improvements in clinical pharmacy clerkship program in undergraduate pharmacy education: the evidence based improvement (EBI) initiative. 2014;2(4):270-5.

16.	Sung Y, Choi JJJotACoN. Protein supplement usage among Male University students: comparisons between current and previous users. 2018;37(2):127-32.

17.	Solak BB, Akin NJJoFS, Engineering. Health benefits of whey protein: a review. 2012;2(3):129.

18.	Kiecolt-Glaser JK, Belury MA, Andridge R, Malarkey WB, Glaser RJB, behavior,, immunity. Omega-3 supplementation lowers inflammation and anxiety in medical students: a randomized controlled trial. 2011;25(8):1725-34.

19.	Biesalski HK, Tinz JJN. Multivitamin/mineral supplements: Rationale and safety. 2017;36:60-6.

20.	Gardiner P, Sarma DN, Dog TL, Barrett ML, Chavez ML, Ko R, et al. The state of dietary supplement adverse event reporting in the United States. 2008;17(10):962-70.

21.	Naqvi AA, Ahmad R, Zehra F, Yousuf R, Kachela B, Nehal Nadir MJJoDS. Dietary supplement use among students of pharmacy colleges in the City of Karachi, Pakistan: prevalence, opinions, and attitudes. 2019;16(2):166-78.

22.	Zehra F, Naqvi AA, Tasneem S, Ahmad R, Ahmad N, Shamsi AZ, et al. Brand versus generic dispensing trend for ciprofloxacin 500 mg, levofloxacin 500 mg, and moxifloxacin 400 mg (oral dosage forms) among pharmacies of Karachi, Pakistan. 2017;7(2):70.

23.	El Khoury G, Ramadan W, Zeeni NJJoch. Herbal products and dietary supplements: a cross-sectional survey of use, attitudes, and knowledge among the Lebanese population. 2016;41(3):566-73.

24.	Ishihara J, Sobue T, Yamamoto S, Sasaki S, Tsugane SJIJoE. Demographics, lifestyles, health characteristics, and dietary intake among dietary supplement users in Japan. 2003;32(4):546-53.

25.	Labadarios D. The role of micronutrient supplementation in chronic disease. Abstract (suppl). 2002.

26.	Braun M, Venter IJSAJoCN. Use of dietary supplements, and awareness and knowledge of the recommended fruit and vegetable intakes and consumption of health food store customers in the Cape Town city bowl. 2008;21(4):323-30.

**Tables:**

**Table 1: Demographic information of the respondents**

| **Items** | **Subgroups** | **Sample (N)** | **Percentage (%)** |
| --- | --- | --- | --- |
| Age | 18-23 years | 859 | 92.9 |
|  | 24-26 years | 66 | 7.1 |
|  | 27-30years | - | - |
| Study year | 1^st^ year | 173 | 18.7 |
|  | 2^nd^ year | 194 | 21.0 |
|  | 3^rd^ year | 215 | 23.2 |
|  | 4^th^ year | 343 | 37.1 |
| Ethnic origin | Bangladeshi | 925 | 100.0 |
|  | Tribal | - | - |
|  | Non-Tribal Bengali | - | - |
| Current marital status | Married | 40 | 4.3 |
|  | Unmarried | 885 | 95.7 |
|  | Divorced | - | - |
| Residence | Living with family | 757 | 81.8 |
|  | Living alone (University accommodation) | 148 | 16.0 |
|  | Living alone (Self accommodation) | 20 | 2.2 |
| Siblings | Between 1&2 siblings | 422 | 45.6 |
|  | Between 3&5 siblings | 355 | 38.4 |
|  | Between 6&8 siblings | 74 | 8.0 |
|  | More than 8 siblings | 37 | 4.0 |
|  | No siblings | 37 | 4.0 |
|  |  |  |  |
| University type | Private | 823 | 89.0 |
|  | Public | 102 | 11.0 |

**Table 2: Suffering of the respondents from illness (if any)**

| **Items** | **Subgroups** | **Sample (N)** | **Percentage (%)** |
| --- | --- | --- | --- |
| Any major illness | Do not suffer from any illness | 399 | 43.1 |
| Suffer from a major illness | Depression | 134 | 14.5 |
|  | Hypertension | 96 | 10.4 |
|  | Diabetes mellitus | 38 | 4.1 |
|  | Sickle cell anaemia | 54 | 5.8 |
|  | Thalassemia | 54 | 5.8 |
|  | Asthma | 72 | 7.8 |
|  | Rheumatoid arthritis | 36 | 3.9 |
|  | Psoriasis | - | - |
|  | Ulcer | - | - |
|  | Irregular ministration | - | - |
|  | Migraine | 21 | 2.3 |
|  | Obesity | - | - |
|  | Epilepsy | - | - |
|  | Others | 21 | 2.3 |

**Table 3: Reasons for use of dietary supplements**

| **Statements** | **SD, N %** | **MD, N %** | **SLD, N %** | **NA/NOD, N %** | **SLA, N %** | **MA, N %** | **SA, N %** |
| --- | --- | --- | --- | --- | --- | --- | --- |
| Physician recommendations | 123  13.3 | 160  17.3 | 160  17.3 | 180  19.5 | 116  12.5 | 85  9.2 | 101  10.9 |
| General health and well being | 119  12.9 | 163  17.6 | 162  17.5 | 180  19.5 | 112  12.1 | 87  9.4 | 102  11.0 |
| For weight gaining | 114  12.3 | 166  17.9 | 166  17.9 | 184  19.9 | 109  11.8 | 88  9.5 | 98  10.6 |
| For energy source | 122  13.2 | 161  17.4 | 163  17.6 | 181  19.6 | 115  12.4 | 89  9.6 | 94  10.2 |
| Immune booster | 119  12.9 | 166  17.9 | 159  17.2 | 178  19.2 | 119  12.9 | 87  9.4 | 97  10.5 |
| Increase performance/sports | 121  13.1 | 157  17.0 | 162  17.5 | 178  19.2 | 120  13.0 | 88  9.5 | 99  10.7 |
| To control hair fall and skin care | 123  13.3 | 156  16.9 | 168  18.2 | 175  18.9 | 116  12.5 | 90  9.7 | 97  10.5 |
| Increase endurance/body building | 121  13.1 | 160  17.3 | 159  17.2 | 180  19.5 | 119  12.9 | 87  94 | 99  10.7 |
| Memory enhancer | 118  12.8 | 162  17.5 | 163  17.6 | 183  19.8 | 113  12.2 | 90  9.7 | 96  10.4 |
| Other reasons(pregnancy-induced anaemia & fatigue) | 120  13.0 | 159  17.2 | 169  18.3 | 180  19.5 | 113  12.2 | 88  9.5 | 96  10.4 |
| No reason mentioned | 124  13.4 | 158  17.1 | 163  17.6 | 182  19.7 | 112  12.1 | 87  9.4 | 99  10.7 |

(SD=strongly Disagree, MD=Moderately disagree, SLD=Slightly disagree, NA/NOD=Neither agree Nor disagree, SLA=Slightly agree, MA=Moderately Agree, SA= strongly agree)

**Table 4: Types of dietary supplement used**

| **Statements** | **SD, N %** | **MD, N %** | **SLD, N %** | **NA/NOD, N %** | **SLA, N %** | **MA, N %** | **SA, N %** | **Total, N %** |
| --- | --- | --- | --- | --- | --- | --- | --- | --- |
| Multivitamins alone or in combination with others | 123  13.3 | 158  17.1 | 164  17.7 | 180  19.5 | 118  12.8 | 86  9.3 | 96  10.4 | 925  100 |
| Ginseng and Gingko biloba | 120  13.0 | 158  17.1 | 168  18.2 | 180  19.5 | 115  12.4 | 86  9.3 | 98  10.6 | 925  100 |
| Omega 3 fatty acid | 124  13.4 | 162  17.5 | 163  17.6 | 180  19.5 | 113  12.2 | 86  9.3 | 97  10.5 | 925  100 |
| Whey protein | 120  13.0 | 158  17.1 | 166  17.9 | 181  19.6 | 114  12.3 | 85  9.2 | 101  10.9 | 925  100 |
| Calcium | 127  13.7 | 161  17.4 | 161  17.4 | 177  19.1 | 113  12.2 | 89  9.6 | 97  10.5 | 925  100 |
| Other supplements (prescription and natural products) | 125  13.5 | 159  17.2 | 168  18.2 | 179  19.4 | 110  11.9 | 86  9.3 | 98  10.6 | 925  100 |
| No supplements used | 362  39.1 | 130  14.1 | 121  13.1 | 121  13.1 | 71  7.7 | 55  5.9 | 65  7.0 | 925  100 |

**Table 5: Cost of dietary supplements per month in Taka**

| **Cost/Month** | **Sample (N)** | **Percentage (%)** |
| --- | --- | --- |
| BDT 10-500 | 817 | 88.3 |
| BDT 501-1000 | 74 | 8.0 |
| BDT 1001-5000 | 34 | 3.7 |
| BDT 5001-10000 | - | - |

**Table 6: Adverse reactions experienced from dietary supplement**

| **Adverse reactions experienced from dietary supplement** | **Variables** | **Sample (N)** | **Percentage (%)** |
| --- | --- | --- | --- |
| Did you experience any adverse reactions? | Yes | 295 | 31.9 |
|  | No | 546 | 59.0 |
| If yes, what were they? | Nausea, vomiting & diarrhoea | 75 | 8.1 |
|  | Confusion, headaches & vertigo | 9 | 1.0 |
|  | Hair fall | - | - |
|  | Rapid weight gain | - | - |
|  | Others | - | - |

**Table 7: Opinions and attitudes of pharmacy students regarding dietary supplement use**

| **Statements** | **SD, N %** | **MD, N %** | **SLD, N %** | **NA/NOD, N %** | **SLA, N %** | **MA, N %** | **SA, N %** | **TotalN %** |
| --- | --- | --- | --- | --- | --- | --- | --- | --- |
| It prevent chronic illness if used regularly | 105  11.4 | 174  18.8 | 167  18.1 | 186  20.1 | 101  10.9 | 89  9.6 | 103  11.1 | 925  100 |
| Safe with minimal risk of adverse effects | 109  11.8 | 166  17.9 | 160  17.3 | 184  19.9 | 111  12.0 | 90  9.7 | 105  11.4 | 925  100 |
| Essential for everyone regardless of age | 102  11.0 | 173  18.7 | 166  17.9 | 185  20 | 101  10.9 | 90  9.7 | 108  11.7 | 925  100 |
| Prevents cancer | 109  11.8 | 166  17.9 | 160  17.3 | 183  19.8 | 109  11.8 | 92  9.9 | 106  11.5 | 925  100 |
| Important for health and general well-being | 103  11.1 | 168  18.2 | 177  19.1 | 187  20.2 | 97  10.5 | 89  9.6 | 104  11.2 | 925  100 |
| Use only as per physician recommendation/ harmful if not used properly | 111  12.0 | 166  17.9 | 160  17.3 | 183  19.8 | 113  12.2 | 90  9.7 | 102  11.0 | 925  100 |
| Necessary for all ages | 130  14.1 | 204  22.1 | 153  16.5 | 179  19.4 | 92  9.9 | 82  8.9 | 85  9.2 | 925  100 |

**Table 8: Dietary supplements are good for health**

| **Items** | **Sample(N)** | **Percentage (%)** |
| --- | --- | --- |
| Agree | 749 | 81.0 |
| Disagree | 37 | 4.0 |
| Do not know | 139 | 15.0 |

**Table 9: Do you personally recommend use of dietary supplements to others**

| **Items** | **Sample (N)** | **Percentage (%)** |
| --- | --- | --- |
| Yes, I always recommend | 493 | 53.3 |
| Yes, only when doctors recommend | 342 | 37.0 |
| Not at all | 90 | 9.7 |

**Table 10: Dietary supplement product information**

| **Items** | **Sample (N)** | **Percentage (%)** |
| --- | --- | --- |
| Brand name | 312 | 33.7 |
| Generic name | 243 | 26.3 |
| Both | 243 | 26.3 |
| Do not know | 127 | 13.7 |

**Table 11: Association of demographic variables with opinions and attitudes**

| Sample (N ) observed (expected)  Dietary supplements are good for health | | | | | |
| --- | --- | --- | --- | --- | --- |
| **Statements** | **Agree** | **Disagree** | **Do not know** | **χ2** | ***p* vale** |
| 1. Age (years)  18-23  24-26 | 695 (695.6)  54 (53.4) | 34 (34.4)  3 (2.6) | 130 (129.1)  9 (9.9) | 0.151 | 0.927 |
| 2. Study year  1^st^ year  2^nd^ year  3^rd^ year  4^th^ year | 136 (140.1)  157 (157.1)  182 (174.1)  274 (277.7) | 8 (6.9)  8 (7.8)  9 (8.6)  12 (13.7) | 29 (26)  29 (29.2)  24 (32.3)  57 (51.5) | 4.001 | 0.677 |
| 3. Ethnic origin | 749 (749) | 37 (37) | 139 (139) | - | - |
| 4. Current marital status  Married  Unmarried | 38 (32.4)  711 (716.6) | 0 (1.6)  37 (35.4) | 2 (6)  137 (133) | 5.485 | 0.064 |
| 5. Residence  Living with family  Living alone ( University accommodation)  Living alone ( Self accommodation) | 617 (613)  119 (119.8)  13 (16.2) | 28 (30.3)  7 (5.9)  2 (0.8) | 112 (113.8)  22 (22.2)  5 (3.0) | 4.185 | 0.382 |
| 6. Siblings  Between 1&2 siblings  Between 3&5 siblings  Between 6&8 siblings  More than 8 siblings  No siblings | 341 (341.7)  285 (287.5)  61 (59.9)  31 (30.0)  31 (30.0) | 16 (16.9)  13 (14.2)  4 (3.0)  1 (1.5)  3 (1.5) | 65 (63.4)  57 (53.3)  9 (11.1)  5 (5.6)  3 (5.6) | 4.273 | 0.832 |
| 7. University type  Private  Public | 669 (666.4)  80 (82.6) | 33 (32.9)  4 (4.1) | 121 (123.7)  18 (15.3) | 0.617 | 0.735 |
| Do you encourage the use of dietary supplements? | | | | | |
|  | **Yes, always** | **Yes, only when doctor recommend** | **Not at all** |  |  |
| 1. Age (years)  18-23  24-26 | 454 (457.8)  39 (35.2) | 322 (317.6)  20 (24.4) | 83 (83.6)  7 (6.4) | 1.359 | 0.507 |
| 2. Study year  1^st^ year  2^nd^ year  3^rd^ year  4^th^ year | 89 (92.2)  96 (103.4)  117 (114.6)  191 (182.8) | 69 (20.2)  82 (24)  76 (79.5)  115 (126.8) | 15 (16.8)  16 (18.9)  22 (20.9)  37 (33.4) | 5.268 | 0.510 |
| 3. Ethnic origin | 493 (493.0) | 342 (342.0) | 90 (90.0) | - | - |
| 4. Current marital status  Married  Unmarried | 25 (21.3)  468 (471.7) | 12 (14.8)  330 (327.2) | 3 (3.9)  87 (86.1) | 1.428 | 0.490 |
| 5. Residence  Living with family  Living alone ( University accommodation)  Living alone ( Self accommodation) | 411 (403.5)  70 (78.9)  12 (10.7) | 267 (279.9)  67 (54.7)  8 (7.4) | 79 (73.7)  11 (14.4)  0 (1.9) | 7.844 | 0.097 |
| 6. Siblings  Between 1&2 siblings  Between 3&5 siblings  Between 6&8 siblings  More than 8 siblings  No siblings | 227 (224.9)  183 (189.2)  41 (39.4)  21 (19.7)  21 (19.7) | 160 (156.0)  135 (131.3)  29 (27.4)  10 (13.7)  8 (13.7) | 35 (41.1)  37 (34.5)  4 (7.2)  6 (3.6)  8 (3.6) | 13.575 | 0.094 |
| 7. University type  Private  Public | 439 (438.6)  54 (54.4) | 305 (304.3)  37 (37.7) | 79 (80.1)  11 (9.9) | 0.149 | 0.928 |

**Table 12: Multinomial regression analysis**

| Do you personally recommend use of dietary supplements to others? |  | Coefficient | Odds ratio (OR) | 95% confidence interval | |
| --- | --- | --- | --- | --- | --- |
|  |  |  |  | Lower bound | Upper bound |
| Yes, I always recommend | Age  18-23 years  24-26 years | 1.718  −0.018  0^b^ | 0.982  - | 0.425  - | 2.269  - |
| Yes, only when doctors recommend | Age  18-23 years  24-26 years | 1.050  0.306  0^b^ | 1.358  - | 0.555  - | 3.319  - |

**Figure Legends**

**Figure 1: Percentage of participations from various departmental students.**

Here, BBA: Bachelor of Business Administration, EB: Economics and Banking, CSE: Computer Science and Engineering, EEE: Electrical and Electronics Engineering, ELL: English Language and Literature
