# Supplementary material for: Tendencies and attitudes towards dietary supplements use among undergraduate female students in Bangladesh
Source: PLoS One. 2021 Apr 9;16(4):e0249897. doi: 10.1371/journal.pone.0249897 (PMC8034729; doi:10.1371/journal.pone.0249897)
Supplement: Attachment — Submitted filename: Response to Reviewers R1.docx [file pone.0249897.s005.docx]

**Journal Requirements:**

When submitting your revision, we need you to address these additional requirements.

1. Please ensure that your manuscript meets PLOS ONE's style requirements, including those for file naming. The PLOS ONE style templates can be found at

Response:  The manuscript has been formatted according to Plos One style.

2. Please include additional information regarding the survey or questionnaire used in the study and ensure that you have provided sufficient details that others could replicate the analyses. For instance, if you developed a questionnaire as part of this study and it is not under a copyright more restrictive than CC-BY, please include a copy, in both the original language and English, as Supporting Information.

 Response:  The questionnaire has been incorporated as supporting file (S3 File).

3. Thank you for including your ethics statement:

"The survey was permitted to the institutional review committee of International Islamic University Chittagong (ref: P&D-147/13-19).".

Please amend your current ethics statement to confirm that your named institutional review board or ethics committee specifically approved this study.

Once you have amended this/these statement(s) in the Methods section of the manuscript, please add the same text to the “Ethics Statement” field of the submission form (via “Edit Submission”).

For additional information about PLOS ONE ethical requirements for human subjects research, please refer to <http://journals.plos.org/plosone/s/submission-guidelines#loc-human-subjects-research>.

 Response:  The statement has been revised and incorporated in the main text (highlighted in yellow colour). The research has been approved by the institutional review committee named Planning and Development Committee (P&D committee). The research approval letter titled on (*Research approval by the institutional review committee*) has been incorporated as supporting file (S1 File).

4. We note that you have indicated that data from this study are available upon request. PLOS only allows data to be available upon request if there are legal or ethical restrictions on sharing data publicly. For more information on unacceptable data access restrictions, please see <http://journals.plos.org/plosone/s/data-availability#loc-unacceptable-data-access-restrictions>.

 Response:  All data are available within the revised manuscript and supporting files.

In your revised cover letter, please address the following prompts:

a) If there are ethical or legal restrictions on sharing a de-identified data set, please explain them in detail (e.g., data contain potentially sensitive information, data are owned by a third-party organization, etc.) and who has imposed them (e.g., an ethics committee). Please also provide contact information for a data access committee, ethics committee, or other institutional body to which data requests may be sent.

 Response:  There are no ethical and legal restrictions regarding sharing our data. In our data there is no such information that is sensitive to any particular community. The contact information of ethical review committee is *Chairman, Review Committee (Planning and development committee), Department of Pharmacy, International Islamic University Chittagong, Chittagong, Bangladesh-4318*.

b) If there are no restrictions, please upload the minimal anonymized data set necessary to replicate your study findings as either Supporting Information files or to a stable, public repository and provide us with the relevant URLs, DOIs, or accession numbers. For a list of acceptable repositories, please see <http://journals.plos.org/plosone/s/data-availability#loc-recommended-repositories>.

 Response:  All data are available within the manuscript and as supporting files.

We will update your Data Availability statement on your behalf to reflect the information you provide.

5. Please upload a copy of Figure 1, to which you refer in your text on page 8. If the figure is no longer to be included as part of the submission please remove all reference to it within the text.

 Response:   The revised figure has been uploaded.

[Note: HTML markup is below. Please do not edit.]

Reviewers' comments:

Reviewer's Responses to Questions

**Comments to the Author**

1. Is the manuscript technically sound, and do the data support the conclusions?

The manuscript must describe a technically sound piece of scientific research with data that supports the conclusions. Experiments must have been conducted rigorously, with appropriate controls, replication, and sample sizes. The conclusions must be drawn appropriately based on the data presented.

Reviewer #1: Yes

Reviewer #2: No

Reviewer #3: Yes

2. Has the statistical analysis been performed appropriately and rigorously?

Reviewer #1: Yes

Reviewer #2: Yes

Reviewer #3: Yes

3. Have the authors made all data underlying the findings in their manuscript fully available?

The [PLOS Data policy](http://www.plosone.org/static/policies.action#sharing) requires authors to make all data underlying the findings described in their manuscript fully available without restriction, with rare exception (please refer to the Data Availability Statement in the manuscript PDF file). The data should be provided as part of the manuscript or its supporting information, or deposited to a public repository. For example, in addition to summary statistics, the data points behind means, medians and variance measures should be available. If there are restrictions on publicly sharing data—e.g. participant privacy or use of data from a third party—those must be specified.

Reviewer #1: Yes

Reviewer #2: No

Reviewer #3: No

4. Is the manuscript presented in an intelligible fashion and written in standard English?

PLOS ONE does not copyedit accepted manuscripts, so the language in submitted articles must be clear, correct, and unambiguous. Any typographical or grammatical errors should be corrected at revision, so please note any specific errors here.

Reviewer #1: Yes

Reviewer #2: No

Reviewer #3: Yes

5. Review Comments to the Author

Please use the space provided to explain your answers to the questions above. You may also include additional comments for the author, including concerns about dual publication, research ethics, or publication ethics. (Please upload your review as an attachment if it exceeds 20,000 characters)

**Reviewer #1:**

*Thank you for giving the opportunity to review this article.
The title of the article should be more understandable and self-explanatory.
Please edit the entire manuscript for English grammar and syntax for readability.*

*Abstract:
1. Background is not clear.*

**Response:** We have revised the abstract (highlighted in yellow color).

*2. Methods: Include the study duration.*

**Response:** The duration of the study was three months (March 2018-May 2018), which have been included (highlighted in yellow color).

*3. Avoid abbreviations in the conclusion.*

**Response:** Corrected (highlighted in yellow color).

*Introduction*

*1. The introduction part is too short and didn’t mention about important key points.*

**Response:** We have revised the introduction part. As per reviewer suggestions we also incorporated important key points (highlighted in yellow colour).

*2. The research question is not discussed and formulated with suitable references.*

**Response:** The authors specially developed the research question. It consists of several multiple-choice questions. The questionnaire was further polished by a group of specialists led by two assistant professors. We have explained the question formulation method is written in the revised version of the manuscript according to the reviewer's comments (highlighted in yellow colour).

A pilot study (100 students) was performed to confirm the questionnaire's reliability and validity by using different approaches. We distributed the questionnaire amongst students to get their feedback regarding the understanding and clarity of all questions. The questionnaire was then reviewed by experts in related fields and other expert colleagues within the university. We also asked external reviewers to provide their feedback and opinion in developing/improving the questionnaire to ensure the reliability of the test and compared the results of our pilot study with the results of similar work done previously. We introduced all necessary expert feedback and suggestions accordingly until we had a final questionnaire used in the present study.

*3. How come this study is differed from reference 7 -9?*

**Response:** The differences are given below:

In reference 7, Lieberman, Harris R., et al. showed dietary supplement use patterns among four United States college students. They consider both male and female respondents. All the respondents received a $10 incentive to complete the survey, and students at Cal State and LSU received a class-based extra credit incentive.

In reference 8 (Spencer, Elsa H et al), the author's main objective was to provide data on medical students' multivitamin and calcium supplement use during medical school. They mostly focused on non-underweight and non-exercising students may be essential targets for messages regarding appropriate and adequate vitamin/mineral use.

In references 9 (Alfawaz et al.), the authors aim to investigate the prevalence of dietary supplement use and its association with sociodemographic/lifestyle characteristics among Saudi female students. Among the participant, 25.5% were married and 74.5% single. Moreover, participants are from undergraduate and graduate level.

In our investigation, the primary focus is to investigate the tendencies and attitudes of Bangladeshi undergraduate female students (age group between 18-23 years) towards dietary supplements.

*4. Mention the aim or objective of the study.*

**Response:** The main aim and objective of this study are appended in the abstract section. Also, it has been quoted here.

Dietary supplements are products that improve individuals' overall health and well-being and reduce the risk of disease. Evidence indicates a rising prevalence of these products worldwide, especially among the age group 18-23 years. The study investigates the tendencies and attitudes of Bangladeshi undergraduate female students towards dietary supplements.

*5. Define the clinical significance of this study in related to researchers, clinicians and patients.*

**Response:** The micronutrient depletion situation in Bangladesh is growing day by day. In Bangladesh, one-fourth of the population is suffering from chronic energy deficiency. Among them, females are more prone to suffer from nutritional deficiencies than men due to various factors such as female reproductive biology, low social status, poverty, and lack of education. This research will provide comprehensive information related to prevalence, user opinions, and attitudes towards DS among undergraduate female students in Chittagong city of Bangladesh. This will lead the researcher to find out how demographic and lifestyle factors, such as monthly cost, educational status, and lifestyle status, correlate positively with dietary supplements consumption.

*Methods
6. Why the search is limited with female students?*

**Response:** Females are more predisposed to suffer from nutritional deficiencies than men due to various factors such as female reproductive biology, low social status, poverty, and lack of education. Socio-cultural practices and inequalities in domestic job habits may also increase the possibility of malnutrition for females. Another reason is also is; no such study has been conducted for Bangladeshi female undergraduate students. Our future goal is to lead similar types of study amidst the male population.

*7. The duration of search?*

**Response:** The duration was: March 2018 to May 2018.

*8. The selection criteria should be more specific (inclusion and exclusion).*

**Response:** The selection criteria are included in the study design and duration section.

*9. How the sample size was calculated - explain clearly?*

**Response:** From seven private and public universities, ten departments were selected by using a lottery. A sample size of 664 was calculated by using Raosoft, Inc. However, by hypothesizing of getting a low number of participants with dietary supplements' previous experience and considering the number of drops out, a total of 1000 undergraduate female students with even ID number was selected randomly from odd and even students ID. Though only 75 female students did not match the study's inclusion criteria, and finally, 925 female students were interviewed and included in the study.

*10. Mention the procedure, reliability and validity of DSQ.*

**Response:** We have added the procedure, reliability, and validity of DSQ in the revised manuscript under the Research instrument development section (highlighted in yellow color).

A pilot study (100 students) was performed to confirm the questionnaire's reliability and validity by using different approaches. We distributed the questionnaire amongst students to get their feedback regarding the understanding and clarity of all questions. The questionnaire was then reviewed by experts in related fields and other expert colleagues within the university. We also asked external reviewers to provide their feedback and opinion in developing/improving the questionnaire to ensure the reliability of the test and compared the results of our pilot study with the results of similar work done previously. We introduced all necessary expert feedback and suggestions accordingly until we had a final questionnaire used in the present study.

*11. Software used for statistical analysis.*

**Response:** The data were analyzed by statistical software Statistical Package for Social Science (SPSS, version 22.0, IBM Corporation, NY).

*Discussion*

*12. Refine the conclusion according to the objective of your study.*

**Response:** According to reviewers remarked we have revise the entire conclusion section matched with the objective of our study (highlighted in yellow color).

**Reviewer #2:**

Comments are found in the attached file, using side comments/track changes

**Response:** Comments found in track change are revised as per your suggestions. For further information please check the revised manuscript.

*Major comments:*

*The title is not representing the content. While all the recruited participants were females, the authors titled the study with "female students are more inclined", more than whom?*

**Response:** Title has been revised.

*The authors concluded that health-related departments were more prevalent with DS use, while nothing mentioned in the 12 tables about the academic departments.*

**Response:** In Table 7, we have summarized the opinions and attitudes of pharmacy students regarding dietary supplement use (highlighted in yellow color).

*The study objectives and specific aims are not clearly mentioned. The 12 tables are dissecting different aspects from the mentioned aim of the study.*

**Response:** According to the reviewers suggestion we have added the objectives and specific aims in the abstract and materials and methods section (highlighted in yellow color).

**Aim**

The study investigates the tendencies and attitudes of Bangladeshi undergraduate female students towards dietary supplements (DS).

**Purposes of the study**

The primary purpose of our survey was to explore the dynamics of using dietary supplements by the undergraduate females, involving the respondent's choice, age, discipline, nature of the institution. The survey was directed among several age ranges of 18-23, 24-26, and 27-30 years. The cause of the ordination is the education period. The inclusion of undergraduate students usually occurs in 18 years, and the duration varies from 4-5 years in different disciplines. The second principal objective was to inquire about DS's differences with the variation of demography, particular opinion, attitudes, disciplines, and ages. Expense regarding dietary supplement usage was recorded and indicated by the BDT (Bangladeshi Taka).

*The conclusions are not supported by the presented data. The authors dismissed any article related to DS use in Bangladesh in their discussion.*

**Response:** According to reviewers remarked we have revise the entire conclusion section matched with the objective of our study (highlighted in yellow color).

**Reviewer #3:**

Paper titled (Tendencies and attitudes of young students towards dietary supplements in Bangladesh: female students are more inclined) by Rahman et al., studied the gender difference towards the dietary supplements in a national area in Bangaldish.

I appreciate such simple and hubmble trial, the study is interestingly written with correct statistical analysis. I recommend publication after a minor revision:
If the authors can discuss and metnion the specific type of supplements that are commonly used in this study in relation to the age ranges.

**Response:** Thank you very much for your valuable remarks. The present study investigates the tendencies and attitudes of Bangladeshi undergraduate female students towards dietary supplements. Several classes (Ginseng and Gingko biloba, Omega 3 fatty acid, Whey protein, Calcium, Natural products) supplements have been analyzed in this study. The specific type of supplements commonly used in this study has been mentioned in table 4.

6. PLOS authors have the option to publish the peer review history of their article ([what does this mean?](https://journals.plos.org/plosone/s/editorial-and-peer-review-process#loc-peer-review-history)). If published, this will include your full peer review and any attached files.


If you choose “no”, your identity will remain anonymous but your review may still be made public.


**Do you want your identity to be public for this peer review?** For information about this choice, including consent withdrawal, please see our [Privacy Policy](https://www.plos.org/privacy-policy).

Reviewer #1: No

Reviewer #2: No

Reviewer #3: **Yes:** Sawsan A Zaitone

[NOTE: If reviewer comments were submitted as an attachment file, they will be attached to this email and accessible via the submission site. Please log into your account, locate the manuscript record, and check for the action link "View Attachments". If this link does not appear, there are no attachment files.]

While revising your submission, please upload your figure files to the Preflight Analysis and Conversion Engine (PACE) digital diagnostic tool, <https://pacev2.apexcovantage.com/>. PACE helps ensure that figures meet PLOS requirements. To use PACE, you must first register as a user. Registration is free. Then, login and navigate to the UPLOAD tab, where you will find detailed instructions on how to use the tool. If you encounter any issues or have any questions when using PACE, please email PLOS at [figures@plos.org](mailto:figures@plos.org). Please note that Supporting Information files do not need this step.
