# Supplementary material for: Tendencies and attitudes towards dietary supplements use among undergraduate female students in Bangladesh
Source: PLoS One. 2021 Apr 9;16(4):e0249897. doi: 10.1371/journal.pone.0249897 (PMC8034729; doi:10.1371/journal.pone.0249897)
Supplement: Attachment — Submitted filename: Response to Reviewer R2_Final.docx [file pone.0249897.s006.docx]

**Response to editorial and reviewer’s comments**

**Dear Editor**

Thank you so much for your tremendous effort for having the manuscript reviewed. We have found both the editorial and reviewers comments highly impactful for improving the manuscript. We have addressed all the issues raised by reviewers and editorial office. Please scroll down to see the reviewer’s response below:

**Journal Requirements:**

Please review your reference list to ensure that it is complete and correct. If you have cited papers that have been retracted, please include the rationale for doing so in the manuscript text, or remove these references and replace them with relevant current references. Any changes to the reference list should be mentioned in the rebuttal letter that accompanies your revised manuscript. If you need to cite a retracted article, indicate the article’s retracted status in the References list and also include a citation and full reference for the retraction notice.

***Author’s Response***: Authors would like to thank the editor for such concern over retracted articles. We, indeed, have not cited any retracted article in our manuscript. Additionally, the manuscript has been formatted according to Plos One style.

**Comments to the Author**
**1. If the authors have adequately addressed your comments raised in a previous round of review** and you feel that this manuscript is now acceptable for publication, you may indicate that here to bypass the “Comments to the Author” section, enter your conflict of interest statement in the “Confidential to Editor” section, and submit your "Accept" recommendation.

Reviewer #1: All comments have been addressed

Reviewer #3: All comments have been addressed

**2. Is the manuscript technically sound and do the data support the conclusions?**
The manuscript must describe a technically sound piece of scientific research with data that supports the conclusions. Experiments must have been conducted rigorously, with appropriate controls, replication, and sample sizes. The conclusions must be drawn appropriately based on the data presented.

Reviewer #1: Yes

Reviewer #3: Yes

**3. Has the statistical analysis been performed appropriately and rigorously?**

Reviewer #1: Yes

Reviewer #3: No

***Author’s Response****:* Authors have rechecked the probable inadequacy of statistical analysis and addressed in a right manner.

**4. Have the authors made all data underlying the findings in their manuscript fully available?**
The [PLOS Data policy](http://www.plosone.org/static/policies.action#sharing) requires authors to make all data underlying the findings described in their manuscript fully available without restriction, with rare exception (please refer to the Data Availability Statement in the manuscript PDF file). The data should be provided as part of the manuscript or its supporting information, or deposited to a public repository. For example, in addition to summary statistics, the data points behind means, medians and variance measures should be available. If there are restrictions on publicly sharing data—e.g. participant privacy or use of data from a third party—those must be specified.

Reviewer #1: Yes

Reviewer #3: No

***Author’s Response****:* In response to the reviewer 3, authors would like to show the gratitude to the reviewer for his keen observation. By the way, authors have ensured to make all the data available in the main manuscript and supporting files of the revised manuscript.

**5. Is the manuscript presented in an intelligible fashion and written in standard English?**
PLOS ONE does not copyedit accepted manuscripts, so the language in submitted articles must be clear, correct, and unambiguous. Any typographical or grammatical errors should be corrected at revision, so please note any specific errors here.

Reviewer #1: Yes

Reviewer #3: No

***Author’s Response****:* Authors are happy to denounce that the manuscript has been crosschecked by all the authors for any grammatical or linguistic error. However, authors will appreciate very particular suggestion or recommendation for any mistake/change.

**6. Review Comments to the Author**

Please use the space provided to explain your answers to the questions above. You may also include additional comments for the author, including concerns about dual publication, research ethics, or publication ethics. (Please upload your review as an attachment if it exceeds 20,000 characters)

**Reviewer #1: Reviewer comments:**

Thank you for giving the opportunity to review this article.

**Abstract:**
1. Mention clearly in the conclusion, what prevalence? DS

***Author’s Response:*** The sentence has been rephrased accordingly.

**Methods**
2. Include the selection criteria of participants in detail.

***Author’s Response:*** Authors wish to thank the reviewer for insightful suggestion. The inclusion criteria for participants were:

i. Undergraduate female students;
ii. Willingness to participate in the study,
iii. Age >18 years;
iv. Ability to understand English or Bengali, and
v. Previous experiences for dietary supplements.

**3. Include the reliability and validity of outcome measures with references.**

***Author’s Response****:* Very appreciating comments indeed from the reviewer. We have included the reliability and validity of outcome measures in the revised manuscript under the Research instrument development section (highlighted in yellow color). We have supplemented the final questionnaire for your kind consideration.

A pilot study (100 students) was performed to confirm the questionnaire's reliability and validity using different approaches. We distributed the questionnaire amongst students to get their feedback regarding the understanding and clarity of all questions. The questionnaire was then reviewed by experts in related fields and other expert colleagues within the university. We also asked external reviewers to provide their feedback and opinion in developing/improving the questionnaire to ensure the reliability of the test and compared the results of our pilot study with the results of similar work done previously. We introduced all necessary expert feedback and suggestions accordingly until we had a final questionnaire used in the present study.

**4. Mention who has extracted the data and their experience and qualifications.**

***Author’s Response:***  Data were extracted by the well-trained final year B. Pharm/M.Pharm research fellows under the direct supervision of a Professor of Biochemistry, working since 16 years (PD from Japan), who has a long expertise on the functional food and alternative medicine filed. Before data collection, they have attended seven days of hands-on training on the dietary supplement questionnaire (DSQ). The whole research work was assisted by two Assistant Professors (having eight to ten years research experience) of the department of pharmacy, International Islamic University Chittagong, Bangladesh.

Discussion
**5. The discussion part should discuss the relation between the DS and its effects with latest references.**

***Author’s Response:*** Very thoughtful suggestion, thank you so much dear reviewer. Discussion part has been revised very carefully in accord with the suggestion of the reviewer. The relation between DS and its effects has been well-discussed and changes of the revised manuscript in this regard have been have been highlighted using a yellow text-highlighter (…..).

**6. Add the clinical significance of this article over the participants and researchers.**

***Author’s Response:*** We appreciate such a reader-demanded suggestion from the reviewer. Indeed, the status of micronutrient depletion in Bangladesh is growing over the days. In Bangladesh, one-fourth of the population has been suffering from chronic energy deficiency. Among them, females are more prone to suffer from nutritional deficiencies than male because of various factors such as lack of awareness among females, their abnormalities in reproductive biology, lack of adequate knowledge, social and economic discrimination in prioritization for females. This research will create an extensive observation and perception on the prevalence of dietary supplements, necessity and trending of supplements by the educated females, their opinions over experiences and overall attitudes towards the use of DS among undergraduate female students in Chittagong, Bangladesh. This will lead the researcher to determine how demographic and lifestyle issues such as monthly cost, educational status and life-status expenditure are correlated with dietary supplement consumption.

**Reviewer #3:** Paper titled (Trends and attitudes towards the use of dietary supplements by female university students in Bangladesh)

Spearman’s rank correlation was employed. Regression analysis was conducted to determine different relationships between DS use and demographic characteristics of students:
This reviewer cannot find the figure in which a regression analysis was done, kindly add to the paper
Stat analysis in tables, I find it mandatory to stat analyze the percentages to explore the real differences between groups and conclude better on the results.

- Spearman’s rank correlation was employed.

***Author’s Response:*** Thank you for your worthy remarks. We are confirming through several discussions with our senior statistician that **chi-square test** is the best befitting analytical tool for our study and we accomplished that to interpret the associations between demographic information and opinions/attitudes our study. We are truly sorry and apologizing for misdirecting the analytical approach on stating Spearman's rank correlation which was not included indeed. We hope the reviewers will accept the author’s clarification. We are again thanking the reviewer for keen observation.

- Regression analysis was conducted to determine different relationships between DS use and demographic characteristics of students. This reviewer cannot find the figure in which a regression analysis was done, kindly add to the paper.

***Author’s Response:*** Many thanks for this comment to improve the quality of our manuscript. In our study, we actually performed **the multinomial logistic regression**, which is relevant for this study. The findings of the multinomial logistic regression analysis are **presented in Table 12.** Addition/changes in the revised manuscript according to the reviewer's comments have been highlighted in the text using a yellow color highlighter (…..).

- Stat analysis in tables, I find it mandatory to stat analyze the percentages to explore the real differences between groups and conclude better on the results.

***Author’s Response:*** Thank you for your comments. In our study's statistical analysis, the **percentages** to explore the real differences between groups were performed and presented in **Tables 1 to 10.** Addition/changes in the revised manuscript according to the reviewer's comments have been highlighted in the text using a yellow color highlighter (…..).
